# Practitioners' Bookshelf

**Published:** 1892-11-12

**Authors:** 


### PRACTITIONERS' BOOKSHELF.

### DISSECTIONS ILLUSTRATED.*

No one can doubt that Mr. Brodie has done a good and a useful work in the publication of his "illustrated dissections" of the human body. He describes his work as a "graphic handbook for students of human anatomy," and the avowed aims of the author have been fulfilled to the letter, namely, that of producing for the impecunious but exacting medical student a portable volume of plates, certainly inferior to those of Ellis in no respect except that of size, and at the same time of providing the busy practitioner with, if we may employ the term, "life-like" representations of the dissections with which he was once familiar in the dissecting-room, but to which he has no longer convenient access.

The folio which we have before us is the first of a series of four, and it deals with the anatomy of the upper limb, illustrating the most familiar dissections of the part with 17 coloured plates and ten diagrams; the whole volume numbers only some 35 pages in all, so that the student is not cumbered with any unnecessary letterpress. Of such high merit are the plates of this, the first volume, that the publication of the three remaining numbers of the series will be awaited with impatience by all students of anatomy. When the work is so good and accurate both from an artist's and from a practical dissector's point of view, it may perhaps seem hypercritical and carping to quarrel with so small a detail as the tints by which the nerves and muscles are delineated; we cannot, however, help thinking that if the muscles had been red and the nerves white, as they are seen on the operating and on post-mortem tables, instead of the reddish-brown and grey of the mummified subject in a London disecting-room, the interests of the dissector would have been better studied, the pictures more diagramatic, and the effect no less artistic. Again, the uncoloured diagrams just fall short of being really first-class. A diagram to be good should be plain and capable of mental reproduction in the brain of the student, but the diagram of the "brachial plexus." as represented on page four, with its labyrinthine and weird tangle of nerves, and its maze of minute letterpress, so far from being capable of reproduction in the brain of the sober student, could exist alone in the disordered fancies of the victim of delirium or nightmare.

* "A Graphic Handbook for Students of Human Anatomy." By C. Gordon Brodie, F.R.C.S., with plates by Percy Highley.

